# L-shaped association of low-density lipoprotein cholesterol with all-cause and cardiovascular mortality in cancer survivors: a population-based cohort study

**DOI:** 10.3389/fcvm.2025.1593824

**Published:** 2025-09-15

**Authors:** Bowen Hou, Yali Hu, Hairong Wang, Huang Zhang, Xingyu Gao, Ying Cui, Yilin Zhao, Jing Xie, Xiaomei Yu, Lang Wang, Hong Jiang, Lihua Zhu

**Affiliations:** ^1^ Department of Cardiology, Renmin Hospital of Wuhan University, Wuhan, China; ^2^ Cardiovascular Research Institute of Wuhan University, Wuhan, China; ^3^ Hubei Key Laboratory of Cardiology, Wuhan, China; ^4^ Department of Cardiology, Zhongnan Hospital of Wuhan University, Wuhan, China

**Keywords:** cancer survivors, LDL-C levels, population study, mortality, NHANES

## Abstract

**Objectives:**

To evaluate the association between LDL-C levels and all-cause, cardiovascular, and cancer mortality in cancer survivors, and to identify the LDL-C level associated with the lowest mortality risk, using data from NHANES 1999–2018.

**Study design:**

Population-based cohort study.

**Methods:**

We analyzed 1,958 U.S. cancer survivors from the National Health and Nutrition Examination Survey (NHANES) 1999–2018. We used Cox and Fine-Gray model to compare mortality risks across LDL-C quartiles, combined with restricted cubic spline analysis to assess nonlinear relationships, and piecewise linear regression to identify LDL-C inflection points.

**Results:**

During a median follow-up of 7.3 years (681 deaths were recorded), we observed a nonlinear association between LDL-C levels and all-cause/cardiovascular mortality, wherein low levels of LDL-C were associated with an increased mortality risk. The identified optimal LDL-C thresholds were 119 mg/dl for all-cause mortality and 124 mg/dl for cardiovascular mortality. Age and CVD history influenced the association, with a negative linear relationship between LDL-C and all-cause mortality observed in individuals aged under 65 years and those in the primary CVD prevention.

**Conclusions:**

Cancer survivors with low LDL-C levels were correlated with elevated all-cause and CVD mortality risks, particularly in younger patients and those without prior CVD.

## 1 Introduction

With population aging and advances in cancer screening, early detection, and therapeutic strategies, the prognosis and long-term survival of cancer patients have substantially improved, resulting in a rapid increase in the number of cancer survivors (1, 2). However, cancer survivors often face a high burden of chronic health conditions related to the late effects of cancer and its therapies. As life expectancy extends, non–cancer-related mortality—primarily from cardiovascular disease (CVD)—has surpassed cancer-related mortality in this population. Low-density lipoprotein cholesterol (LDL-C), a well-established causal risk factor for CVD, has also been implicated in cancer initiation and progression, making it a primary target in dyslipidemia management for cancer survivors.

Despite its clinical importance, current guidelines provide no specific recommendations for LDL-C management in cancer survivors. Previous studies have mainly focused on the relationship between LDL-C levels and cancer-related prognosis during the treatment period in patients with specific cancer types, rather than in long-term survivors (3, 4). Even among cancer patients, findings on the association between LDL-C and mortality remain controversial. Some studies have suggested that higher LDL-C levels are linked to worse survival outcomes (3, 5), whereas others found no significant association (6–9) or even reported an unexpected inverse association (10), in which elevated LDL-C levels were correlated with improved survival. More recently, a Korean cohort study (4) in newly diagnosed cancer patients reported a nonlinear association, indicating that lower LDL-C levels were associated with higher all-cause mortality risk. In contrast, an analysis of the general population from the NHANES 1999–2014 dataset (11) demonstrated a U-shaped association between LDL-C levels and all-cause mortality, but no significant linear or nonlinear association was observed in the subgroup with a prior history of cancer.

To address these uncertainties, we conducted a population-based study using data from the National Health and Nutrition Examination Survey (NHANES) 1999–2018 to evaluate the association of LDL-C levels with all-cause, cardiovascular, and cancer mortality among cancer survivors. We also aimed to identify the LDL-C level associated with the lowest mortality risk.

## 2 Methods

### 2.1 Study design

We conducted a population-based cohort study using NHANES 1999–2018. The NHANES was approved by the National Center for Health Statistics (NCHS) Ethics Review Board (ERB), and all NHANES participants provided written informed consent. Detailed information about the study design and setting can be obtained from the NHANES website (https://wwwn.cdc.gov/nchs/nhanes/).

### 2.2 Population and outcomes

Of the 136,544 individuals screened, 31,083 completed fasting serum lipid tests. These participants were asked: “Have you ever been told by a doctor or other health professional that you had cancer or a malignancy of any kind?” Those who responded “yes” were classified as cancer survivors, resulting in 2,040 individuals (12). After excluding participants younger than 20 years (*n* = 0), pregnant women (*n* = 9), and those with missing LDL-C data (*n* = 73), the final analytic sample consisted of 1,958 individuals (Supplementary Figure S1).

Linkage to National Death Index (NDI) mortality data included information on mortality status, follow-up time, and leading causes of death. Follow-up commenced from the examination in the Mobile Examination Center (MEC) and continued until death or December 31, 2019. Cause-specific mortality was classified according to the 10th revision of the International Statistical Classification of Diseases and Related Health Problems (ICD–10), including diseases of the heart (I00–I09, I11, I13, I20–I51) and malignant neoplasms (C00–C97).

### 2.3 LDL measurements

In NHANES, LDL-C data were calculated using the Friedewald equation based on laboratory measurements of total cholesterol (TC), high-density lipoprotein cholesterol (HDL-C), and triglycerides (TG):

$$\begin{array}{rl} {}[LDL-cholesterol]\;= & \;\;[total\;cholesterol]\;\text{–}\;[HDL-cholesterol]\\ & \;\text{–}\;[triglycerides/5] \end{array}$$

where [triglycerides/5] is the estimated value of very low-density lipoprotein cholesterol (VLDL-C). NHANES reported LDL-C data for survey participants aged 12 years and older who fasted for at least 8.5 h but less than 24 h before lipid testing. Definitions of other covariables were described in Supplementary Materials**-Covariates.**

### 2.4 Statistical analyses

Participants were grouped based on the quartiles of LDL-C levels. We fitted adjusted Cox regression models and Fine-Gray models to examine the association of LDL-C levels with all-cause and cause-specific mortality. The Fine-Gray model accounted for competing risks by considering other causes of death when analyzing a specific cause of mortality. Restricted cubic spline curves with three knots at the 10th, 50th, and 90th percentiles of LDL-C were used to assess nonlinearity. If the relationship proved nonlinear, we conducted a threshold effect analysis using piecewise linear regression to identify LDL-C inflection points, and applied a two-segment Cox proportional hazards model on either side of these inflection points. The atherosclerotic cardiovascular disease (ASCVD) risk was assessed using the American Heart Association/American College of Cardiology (AHA/ACC) risk estimation tool, based on age, diabetes status, total cholesterol, high-density lipoprotein cholesterol (HDL-C), systolic blood pressure, treatment for hypertension, smoking status, and race. Individuals with a history of ASCVD or LDL-C levels ≥190 mg/dl were directly classified into the high-risk group, while those with LDL-C levels <70 mg/dl were categorized separately as the low LDL-C group. Stratified analyses were performed based on gender, age (<65 years or ≥65 years), race (White or non-White), BMI (<25, 25–30, or ≥30.00), smoking status (current smoker or non-current smoker), cardiovascular diseases, diabetes, hypertension, statin use. Interactions between LDL-C groups defined by inflection points and subgroup variables were assessed using the Cox model. The fully adjusted models accounted for gender, age, race, education level, smoking status, body mass index, systolic blood pressure, eGFR, cardiovascular disease, diabetes, statin use and cancer type.

Multiple imputation using the classification and regression trees (CART) method was performed for variables with missing values, including education level, smoking and drinking status, systolic blood pressure, eGFR, BMI, HbA1c, cardiovascular diseases, hypertension, chronic kidney disease, and statin use. The distributions before and after imputation were comparable. The number and percentage of missing data are provided in Supplementary Figure S2. All analyses were conducted using R version 4.3.3, with two-sided *p*-values less than 0.05 considered statistically significant. Statistical analyses were performed according to CDC guidelines (https://wwwn.cdc.gov/nchs/nhanes/tutorials), and the merging and analysis of data accounted for the NHANES complex survey design to obtain unbiased estimates (Supplementary Materials**—Weights in NHANES**).

### 2.5 Sensitivity analyses

We conducted extensive sensitivity analyses to ensure the robustness of our results. First, we used penalized smoothing splines as an alternative to the restricted cubic splines method. Second, we excluded participants with chronic diseases, including diabetes, cardiovascular disease, and chronic kidney disease. Third, we excluded events occurring in the first, second, and third years of follow-up to minimize reverse causality. Fourth, we analyzed data from NHANES 1999–2014 to ensure that each participant had sufficient follow-up time. Fifth, we excluded individuals with missing data and repeated the primary analysis. Sixth, we excluded cancer survivors whose interval between cancer diagnosis and baseline LDL-C measurement was less than three years, aiming to minimize the potential influence of cancer and its treatments, and assessed the robustness of results in mid- to long-term survivors.

## 3 Result

Among the 1,958 cancer survivors included in the analysis, 916 (representing a weighted 41%) were male, with a median age of 65 years (IQR 53–75). Most participants were non-Hispanic White (1,378; weighted 86%). The median LDL-C level was 113 mg/dl (IQR 90–138). Over a median follow-up of 7.3 years (range 0.2–20.7), 681 deaths (34.8%) occurred, including 206 from cancer and 175 from cardiovascular causes. The median age at most recent cancer diagnosis was 54 years (IQR 40–65). The majority of survivors had only one cancer (90%), while 10.2% had two or more cancers. The median time from the most recent cancer diagnosis to baseline was 7 years (IQR 3–15).

Table 1 presents the characteristics of U.S. cancer survivors by LDL-C quartiles in NHANES 1999–2018. Survivors in the lowest LDL-C quartile (<90 mg/dl) were older (median age 69 years) and had a higher prevalence of comorbidities, including diabetes (19%), cardiovascular disease (29%), hypertension (72%), and chronic kidney disease (24%). They were also more likely to use statins (62%). Gender distribution, age at most recent cancer diagnosis, and number of cancer types varied across LDL-C quartiles: 50% of survivors in the first quartile (Q1) were male, while 69% in the fourth quartile (Q4) were female. Survivors in Q1 had an older age at most recent cancer diagnosis, whereas those in Q3 had a lower prevalence of multiple cancer types.

**Table 1:** Characteristics of U.S. cancer survivors, by LDL-C quartiles, NHANES, 1999–2018.

Characteristic	*N* ^a^	LDL-C Group					*p*-value^c^
		Overall, *N* = 1,958 (100%)^b^	Q1(<90 mg/dl), *N* = 552 (26%)^b^	Q2 (90–113 mg/dl), *N* = 487 (25%)^b^	Q3 (113–138 mg/dl), *N* = 492 (25%)^b^	Q4(≥138 mg/dl), *N* = 427 (25%)^b^	
Gender	1,958						<0.001
*Male*		916 (41%)	306 (50%)	227 (40%)	221 (44%)	162 (31%)	
*Female*		1,042 (59%)	246 (50%)	260 (60%)	271 (56%)	265 (69%)	
Age(years)	1,958	65.0 (53.0, 75.0)	69.0 (59.0, 77.0)	63.0 (50.0, 75.0)	63.0 (54.0, 74.0)	62.0 (53.0, 72.0)	<0.001
Race	1,958						0.057
*Non-Hispanic White*		1,378 (86%)	383 (83%)	343 (85%)	347 (87%)	305 (89%)	
*Non-Hispanic Black*		245 (5.5%)	79 (7.2%)	61 (5.2%)	49 (4.2%)	56 (5.3%)	
*Mexican American*		149 (2.6%)	32 (2.1%)	39 (3.2%)	47 (3.0%)	31 (2.1%)	
*Other Hispanic*		102 (2.8%)	30 (2.9%)	26 (3.5%)	29 (3.2%)	17 (1.6%)	
*Other Race*		84 (3.2%)	28 (4.7%)	18 (3.3%)	20 (2.4%)	18 (2.2%)	
Education level	1,955						0.4
*Less than high school*		445 (15%)	145 (18%)	104 (12%)	114 (16%)	82 (13%)	
*High school or equivalent*		472 (25%)	134 (25%)	118 (25%)	114 (23%)	106 (25%)	
*College or above*		1,038 (61%)	272 (57%)	265 (62%)	263 (62%)	238 (62%)	
BMI(kg/m^2^)	1,919	27.41 (24.07, 32.21)	27.90 (24.02, 33.08)	27.39 (23.68, 32.10)	27.80 (24.20, 32.94)	26.80 (24.13, 31.53)	0.3
Systolic Blood Pressure(mmHg)	1,896	124.7 (112.7, 138.7)	124.7 (113.3, 140.0)	124.7 (112.7, 138.7)	124.7 (112.7, 137.3)	124.0 (113.3, 136.7)	0.9
LDL-C(mg/dl)	1,958	113 (90, 138)	77 (66, 84)	102 (95, 109)	125 (119, 132)	156 (147, 171)	<0.001
HDL-C(mg/dl)	1,958	53 (43, 65)	51 (41, 64)	55 (45, 69)	53 (44, 63)	53 (45, 66)	0.067
TG(mg/dl)	1,958	112 (78, 159)	97 (71, 152)	106 (74, 157)	107 (78, 154)	134 (93, 176)	<0.001
TC(mg/dl)	1,958	195 (170, 223)	154 (137, 166)	183 (171, 195)	204 (194, 215)	244 (228, 262)	<0.001
eGFR[ml/(min*1.73^m^2^)]	1,948	85.50 (70.01, 98.01)	80.73 (64.21, 92.72)	87.72 (73.01, 98.92)	85.66 (71.82, 96.69)	88.75 (74.22, 100.75)	<0.001
HbA1c(%)	1,957	5.60 (5.30, 5.90)	5.70 (5.40, 6.20)	5.50 (5.20, 5.90)	5.50 (5.30, 5.80)	5.50 (5.30, 5.80)	<0.001
Smoker	1,955						0.5
*Current smokers*		294 (16%)	82 (15%)	69 (15%)	73 (17%)	70 (18%)	
*Former smokers*		774 (37%)	239 (41%)	198 (38%)	181 (37%)	156 (33%)	
*Non-smokers*		887 (47%)	230 (45%)	219 (48%)	237 (46%)	201 (49%)	
Drinker	1,860						>0.9
*Heavy drinkers*		209 (12%)	59 (11%)	44 (11%)	55 (14%)	51 (13%)	
*Moderate drinkers*		1,031 (59%)	286 (59%)	257 (59%)	259 (58%)	229 (60%)	
*Non-drinkers*		620 (29%)	178 (29%)	156 (30%)	152 (28%)	134 (27%)	
Diabetes	1,958						<0.001
*Yes*		475 (19%)	203 (31%)	109 (18%)	87 (15%)	76 (14%)	
*No*		1,483 (81%)	349 (69%)	378 (82%)	405 (85%)	351 (86%)	
Cardiovascular Disease	1,945						<0.001
*Yes*		426 (17%)	179 (29%)	97 (16%)	81 (14%)	69 (11%)	
*No*		1,519 (83%)	369 (71%)	388 (84%)	407 (86%)	355 (89%)	
Hypertension	1,933						<0.001
*Yes*		1,257 (59%)	401 (72%)	300 (57%)	287 (52%)	269 (55%)	
*No*		676 (41%)	145 (28%)	182 (43%)	197 (48%)	152 (45%)	
Chronic Kidney Disease	1,944						<0.001
*Yes*		433 (16%)	163 (24%)	101 (15%)	99 (15%)	70 (11%)	
*No*		1,511 (84%)	384 (76%)	380 (85%)	392 (85%)	355 (89%)	
Statin Use	1,914						<0.001
*Yes*		721 (34%)	343 (62%)	194 (38%)	120 (23%)	64 (14%)	
*No*		1,193 (66%)	204 (38%)	275 (62%)	361 (77%)	353 (86%)	
Age at most recent cancer diagnosis (years)	1,940	54 (40, 65)	58 (45, 68)	53 (37, 65)	54 (40, 64)	52 (38, 63)	0.007
Number of cancer types	1,958						0.041
*1*		1,751 (90%)	486 (89%)	441 (92%)	442 (89%)	382 (89%)	
*2*		185 (9.2%)	55 (9.2%)	42 (7.0%)	44 (9.7%)	44 (11%)	
≥*3*		22 (1.0%)	11 (2.2%)	4 (0.6%)	6(1.1%)	1(0.3%)	
Time since most recent cancer diagnosis to baseline (years)	1,940	7 (3, 15)	7 (3, 16)	6 (2, 14)	6 (3, 15)	7 (3, 13)	0.6

^a^ *N* not Missing (unweighted).

^b^ median (interquartile range, IQR) for continuous; *n* (%) for categorical.

^c^ Pearson's X^2^: Rao & Scott adjustment; Design-based KruskalWallis test.

### 3.1 LDL-C and all-cause mortality in cancer survivors

**Figure 1:**
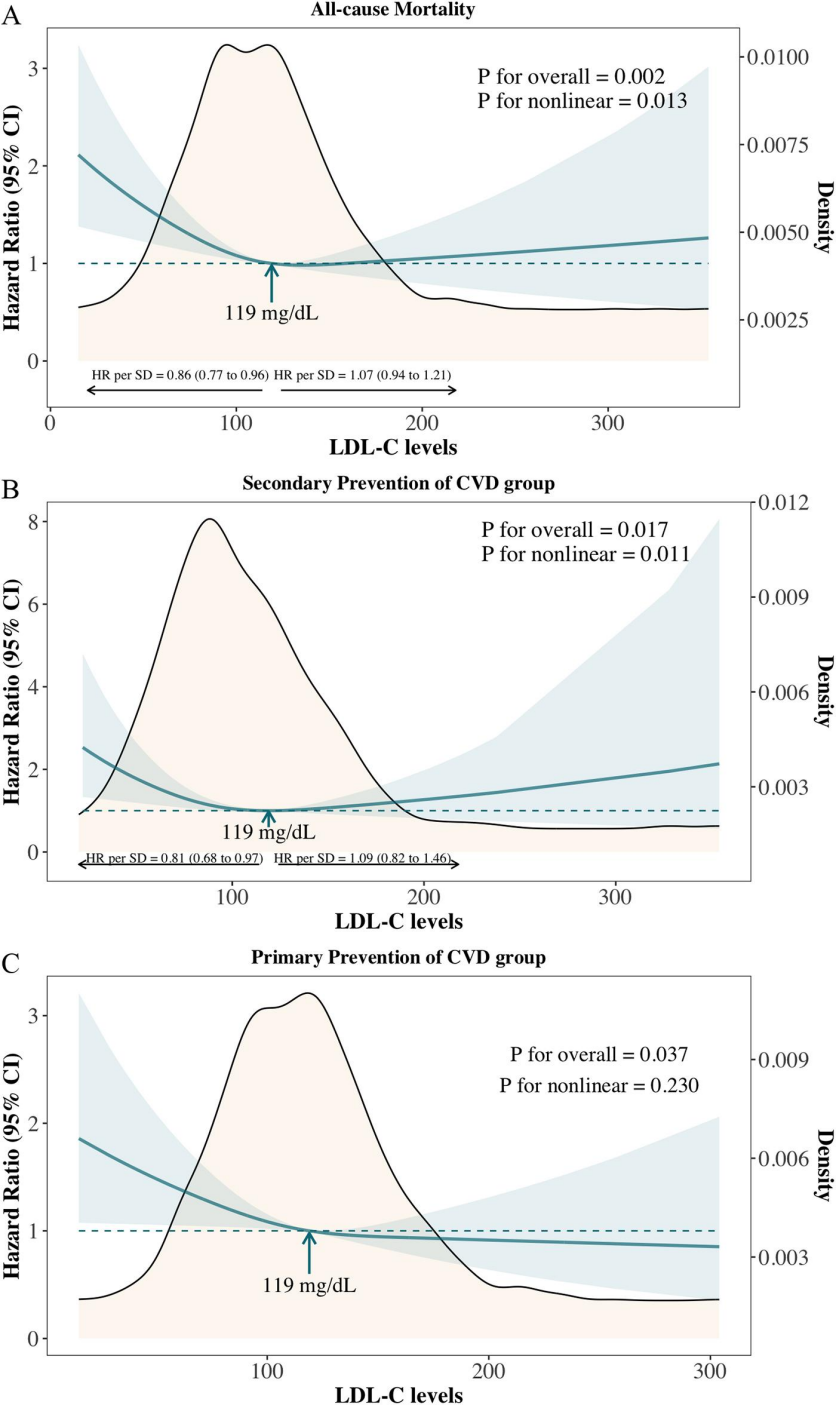
Adjusted hazard ratios and 95% confidence intervals for all-cause mortality according to levels of LDL-C on a continuous scale. **(A)** Overall cancer survivors; **(B)** Primary prevention of cardiovascular diseases (CVD) group: cancer survivors without known CVD; **(C)** Secondary prevention of CVD group: cancer survivors with known cardiovascular diseases.

In overall cancer survivors, a nonlinear association was observed between LDL-C levels and all-cause mortality (*P* for overall association = 0.002; *P* for nonlinear association = 0.015), with low LDL-C levels associated with increased risk (Figure 1A). The inflection point was identified at 119 mg/dl, with an adjusted hazard ratio (aHR) per standard deviation (SD) increase of 0.87 (95% CI: 0.78–0.97) below this level and 1.06 (95% CI: 0.94–1.21) above it. Compared with cancer survivors with levels of LDL-C of 113–138 mg/dl (51st-75th centiles), the multivariable adjusted hazard ratio for all-cause mortality was 1.49 (95% CI: 1.14–1.95) for individuals with levels of LDL-C less than <90 mg/dl (1st-25th centiles) (Supplementary Figure S3).

A similar pattern was observed in the secondary prevention group (cancer survivors with known cardiovascular diseases) (Figure 1B: *P* for overall association = 0.017; *P* for nonlinear association = 0.011). In the primary prevention group (cancer survivors without known cardiovascular diseases), low LDL-C levels were linked to an increased risk of all-cause mortality, though no significant nonlinear association was observed (Figure 1C: *P* for overall association = 0.037; *P* for nonlinear association = 0.230).

### 3.2 LDL-C and cardiovascular, cancer mortality in cancer survivors

**Figure 2:**
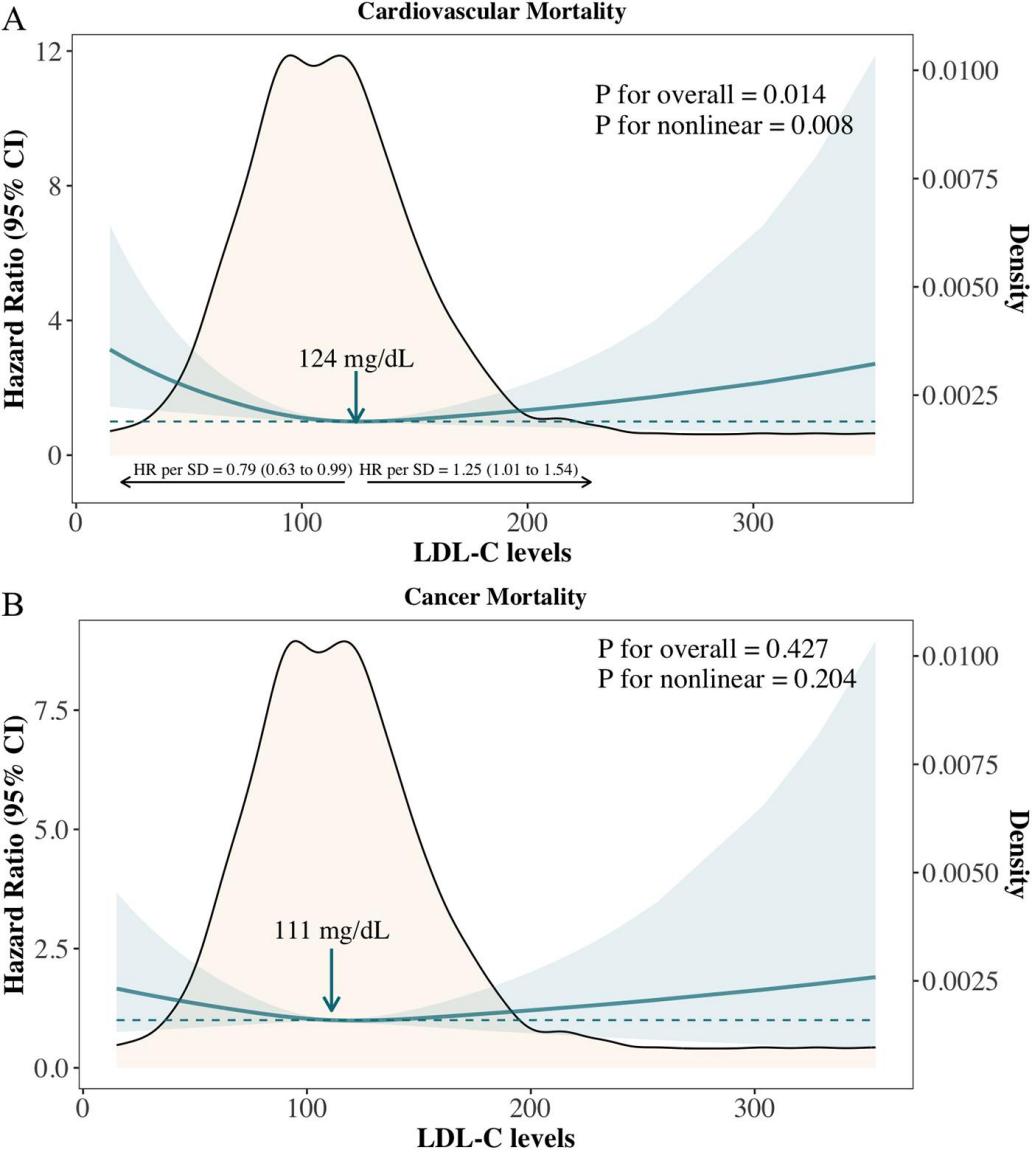
Adjusted hazard ratios for cardiovascular and cancer mortality according to levels of LDL-C on a continuous scale. **(A)** CVD mortality; **(B)** Cancer mortality.

A nonlinear association was also observed between LDL-C levels and cardiovascular mortality in cancer survivors (Figure 2A: *P* for overall association = 0.014; *P* for nonlinear association = 0.008), with the lowest risk identified at 124 mg/dl. The adjusted hazard ratio (aHR) per standard deviation (SD) increase was 0.79 (95% CI: 0.63–0.99) below this level and 1.25 (95% CI: 1.01–1.54) above it. Compared to cancer survivors with LDL-C levels of 113–138 mg/dl (51st–75th percentiles), those with LDL-C < 90 mg/dl (1st–25th percentiles) had significantly higher risks of cardiovascular mortality, with a multivariable cause-specific hazard ratio of 2.40 (95% CI: 1.36–4.25) and a subdistribution hazard ratio of 2.23 (95% CI: 1.36–3.65) (Supplementary Figure S3). No significant association or nonlinear relationship was observed between LDL-C levels and cancer mortality, either on a continuous scale or across quantile groups (Figure 2B and Supplementary Figure S3).

**Figure 3:**
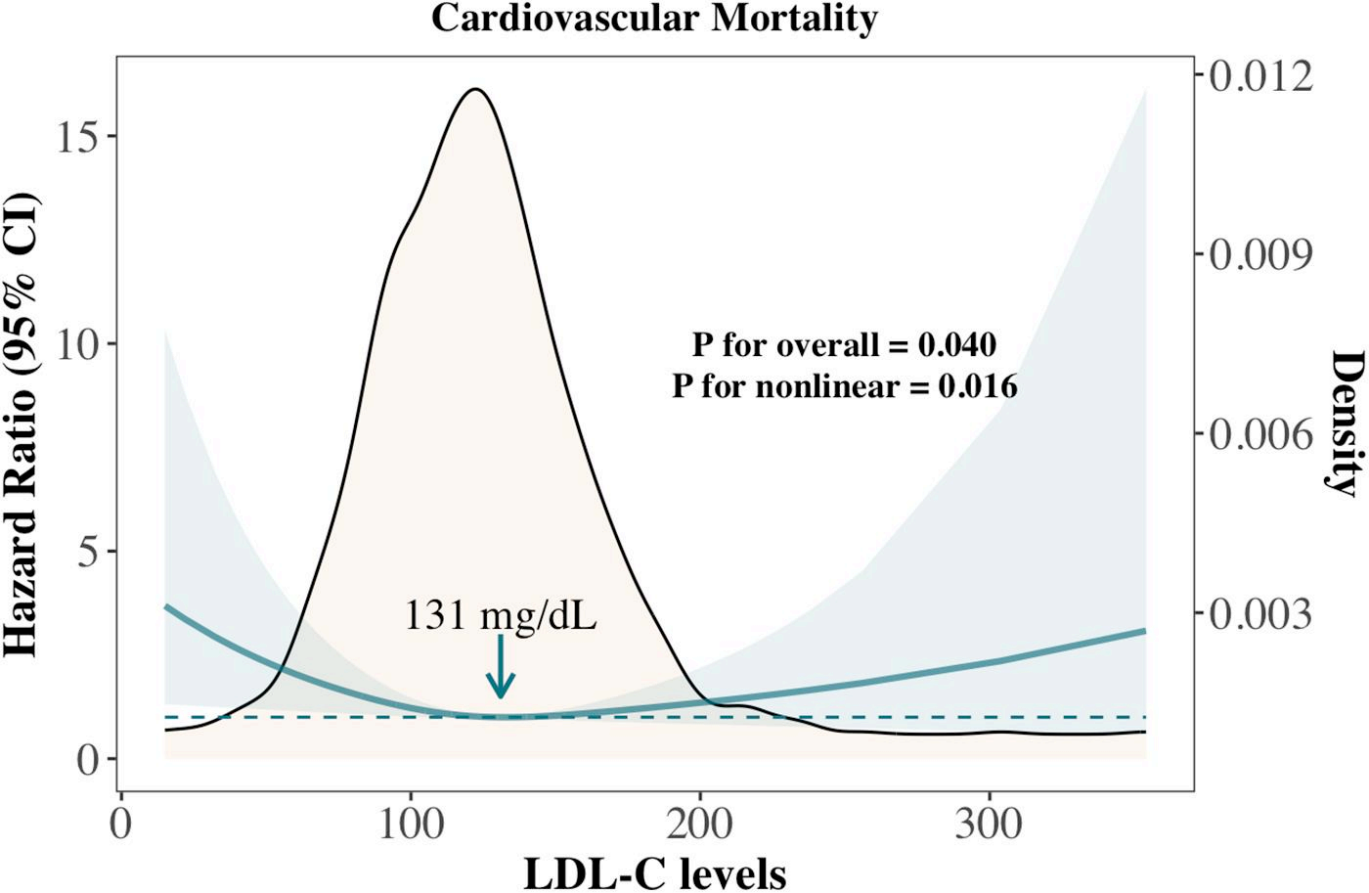
Association between LDL-C and cardiovascular mortality in cancer survivors not treated with statins.

We further compared the cumulative incidence of cardiovascular mortality and cancer mortality among overall cancer survivors, the primary prevention group, and the secondary prevention group (Supplementary Figure S4). Among all cancer survivors, the estimated probability of cancer mortality was higher than that of cardiovascular mortality throughout the follow-up period (Supplementary Figure S4A); however, the cumulative mortality rates of both were nearly identical at the longest follow-up point. In the primary prevention group, the estimated probability of cancer mortality consistently exceeded that of cardiovascular mortality throughout the follow-up period (Supplementary Figure S4B). In contrast, in the secondary prevention group, the estimated probabilities of cancer and cardiovascular mortality were similar during the first 50 months of follow-up. As follow-up continued, the estimated probability of cardiovascular mortality became significantly higher than that of cancer mortality (Supplementary Figure S4C). To avoid the confounding effect of lipid-lowering therapy, we analyzed the association between LDL-C levels and cardiovascular mortality in the subgroup of cancer survivors not receiving statins. The results showed a nonlinear relationship consistent with that observed in the overall survivor cohort (*P* for overall = 0.040, *P* for nonlinear = 0.016), with the lowest risk observed at an LDL-C level of 131 mg/dl (Figure 3).

### 3.3 Subgroup analysis of association between LDL-C and mortality in cancer survivors

**Figure 4:**
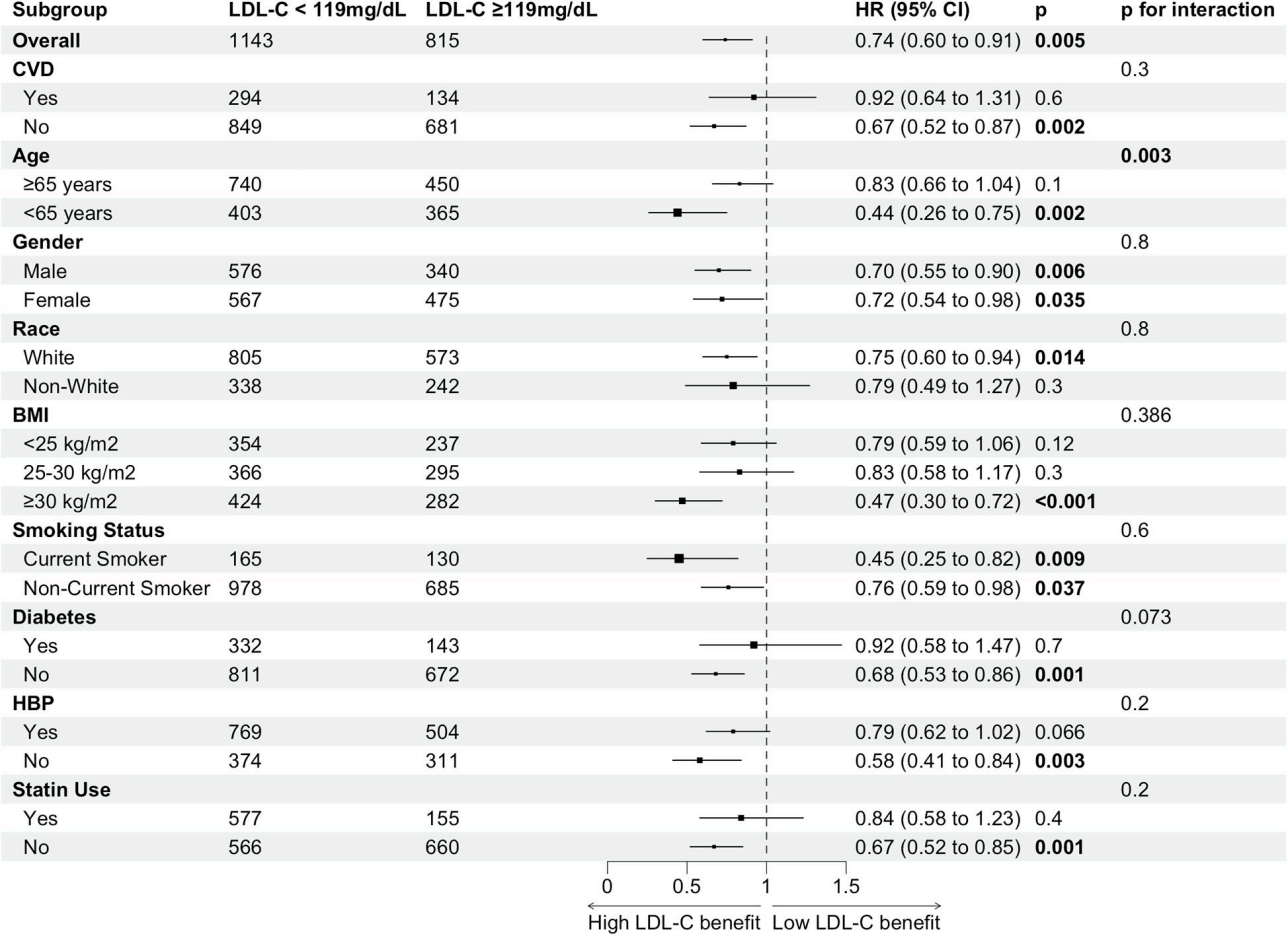
Subgroup analysis of high vs. low LDL-C for all-cause mortality risk in cancer survivors.

We classified cancer survivors into two groups based on the inflection point of LDL-C levels in relation to all-cause mortality: higher LDL-C levels (≥119 mg/dl) and lower LDL-C levels (<119 mg/dl). The association between higher LDL-C levels and a reduced risk of all-cause mortality, compared to lower LDL-C levels, was consistent across most subgroups, with varying magnitudes of adjusted hazard ratios (Figure 4). Confidence intervals were overlapping within and across all subgroups. A significant association between higher LDL-C levels and lower mortality risk was observed across all genders, younger individuals, Whites, those with a BMI ≥ 30 kg/m², current smokers, individuals without cardiovascular disease (CVD), diabetes, and hypertension, as well as those not using statins. The largest numerical differences in hazard ratios for all-cause mortality were observed between older (≥65 years) and younger (<65 years) individuals, with a distinct interaction between LDL-C levels and age (*p* for interaction = 0.003). Additionally, a stronger inverse association between LDL-C and all-cause mortality was observed in current smokers, although this interaction did not reach statistical significance.

### 3.4 Interaction between LDL-C levels and age

**Figure 5:**
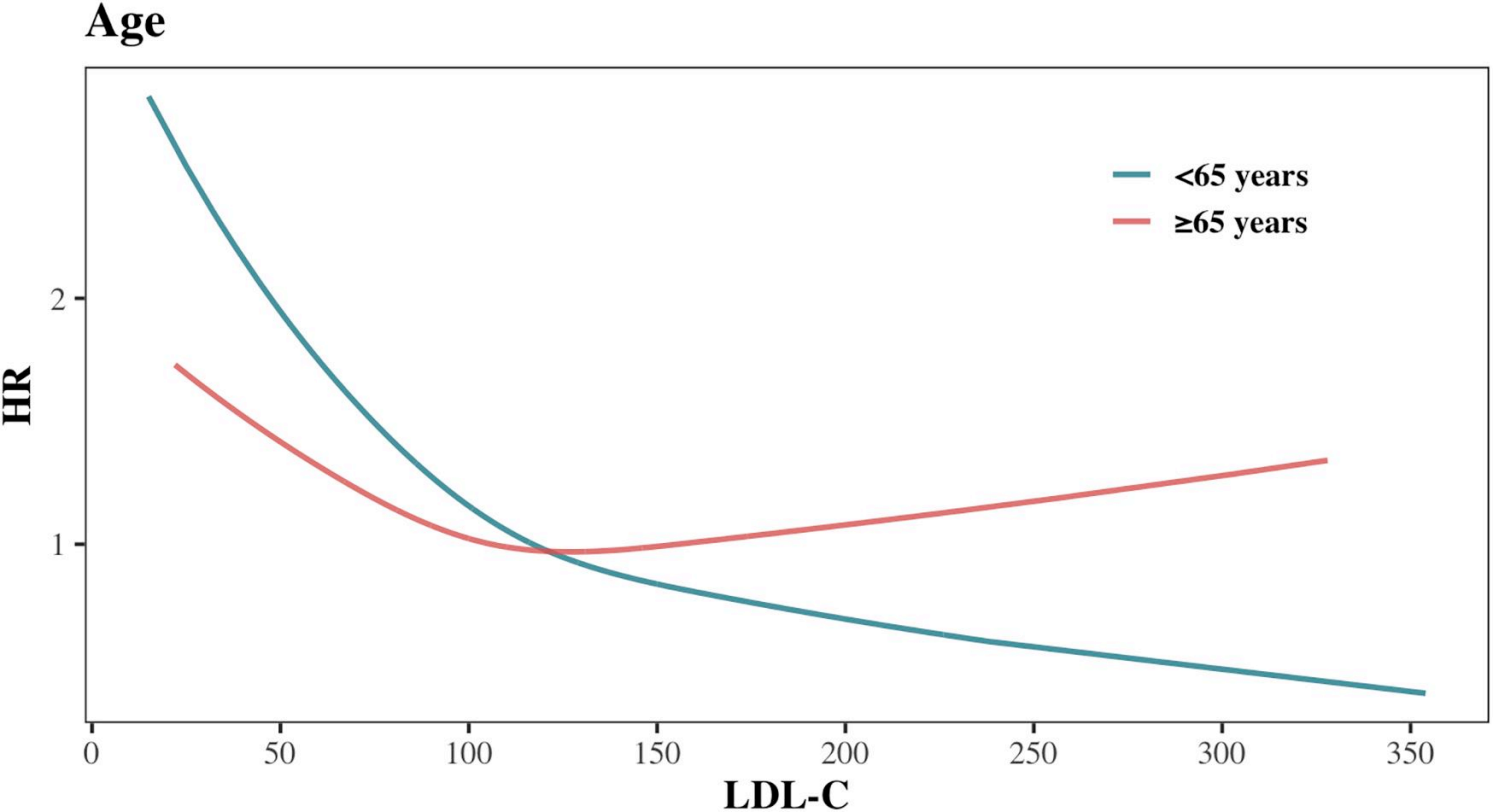
Association between LDL-C levels and all-cause mortality stratified by age.

Figure 5 and Supplementary Figure S5 illustrates the adjusted hazard ratio for all-cause mortality across continuous LDL-C levels by age group. The risk of all-cause mortality decreased with rising LDL-C levels in both age groups. However, beyond 119 mg/dl, the all-cause mortality risk in the <65 years group continued to decrease with increasing LDL-C, while the risk in the ≥65 years group plateaued and even slightly increased. This suggests that the association between high LDL-C levels and all-cause mortality differs across age groups.

**Figure 6:**
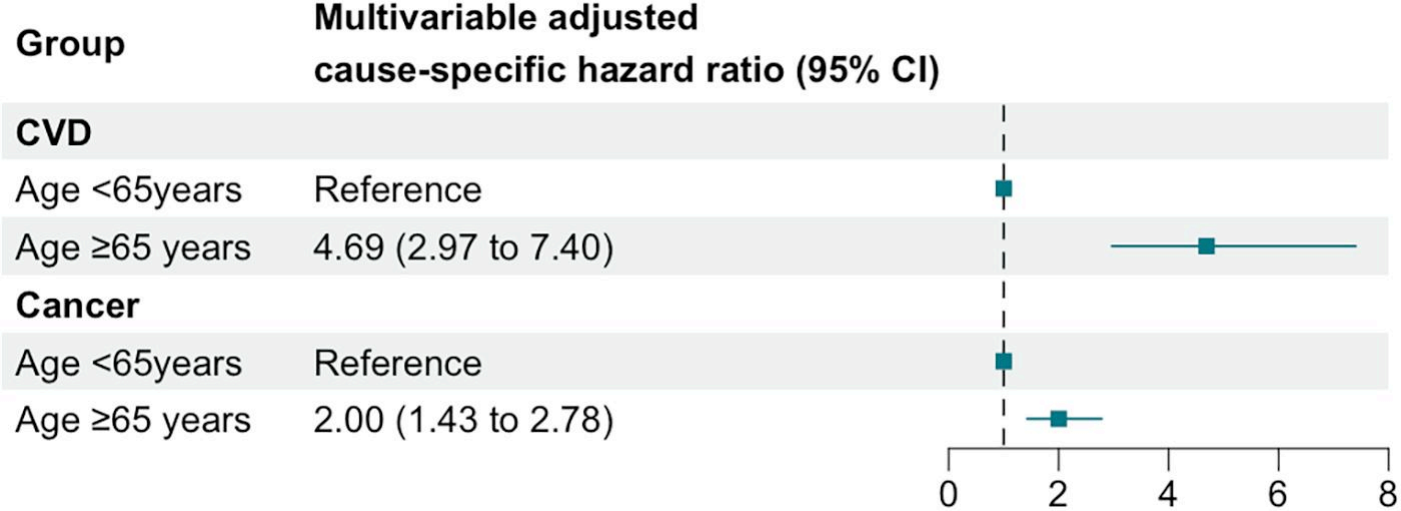
Multivariable adjusted cause-specific hazard ratios and 95% confidence intervals for CVD and cancer mortality across age groups.

To further investigate this difference, we analyzed the distribution of causes of death between the two age groups (Supplementary Figure S6), calculated ASCVD risk in each group (Supplementary Figure S7), and examined the variations in cardiovascular and cancer mortality (Figure 6). Among cancer survivors aged <65 years, cancer was the leading cause of death (*n* = 50, 45.05%), while CVD mortality accounted for only 18.02% (*n* = 20) (Supplementary Figure S6). Conversely, in cancer survivors aged ≥65 years, the proportion of deaths attributed to CVD increased significantly (*n* = 155, 27.19%), nearly equaling the proportion of cancer-related deaths (*n* = 156, 27.37%) (Supplementary Figure S6). ASCVD risk assessment revealed that a higher proportion of cancer survivors in the age ≥ 65 years group were classified as high or moderate risk, whereas the age < 65 years group predominantly fell into low or very low risk categories (Supplementary Figure S7). Results from the Fine-Gray model showed that, compared to the age < 65 years group, the multivariable-adjusted cause-specific hazard ratio (95% CI) for CVD mortality in the age ≥65 years group was 4.69 (2.97–7.40), while for cancer mortality, it was only 2.00 (1.43–2.78) (Supplementary Figure 5). These findings indicate that the relative increase in CVD mortality risk in the age ≥65 years group is substantially greater than the relative increase in cancer mortality risk.

### 3.5 Sensitive analyses

The association of low LDL-C levels with increased all-cause and cardiovascular mortality remained consistent when penalized smoothing splines were used instead of restricted cubic splines (Supplementary Figure S8). To reduce potential reverse causation, we excluded individuals with baseline cardiovascular disease, diabetes, or chronic kidney disease and reanalyzed the relationship between LDL-C and all-cause mortality (Supplementary Figure S9). The results were similar, with the inflection point for LDL-C and all-cause mortality at 117 mg/dl, slightly lower than in the primary analysis. Excluding participants who died within the first, second, or third year of follow-up also yielded consistent findings (Supplementary Figure S9). Additionally, analyses using NHANES 1999–2014 data, ensuring a minimum follow-up of five years, produced comparable results (Supplementary Figure S9). Results remained robust after excluding all participants with missing data. Finally, after excluding cancer survivors diagnosed within three years before baseline, the association between low LDL-C levels and higher all-cause and cardiovascular mortality persisted among mid- to long-term survivors (Supplementary Figure S9).

## 4 Discussion

This population-based study of 1,958 cancer survivors revealed a nonlinear relationship between LDL-C levels and mortality, with low LDL-C (<119 mg/dl for all-cause; <124 mg/dl for cardiovascular mortality) associated with higher risk. Notably, this association persisted in secondary CVD prevention groups, whereas younger survivors (<65 years) and those without prior CVD showed an inverse LDL-C–mortality relationship. Cumulative mortality analyses highlighted competing risks: cancer accounted for most early deaths in primary prevention cohorts, while cardiovascular mortality exceeded cancer-related deaths in secondary prevention groups over extended follow-up.

With population aging and advances in cancer detection and treatment, the number of cancer survivors is rapidly increasing (1, 2). Extended life expectancy has shifted the disease burden toward chronic non-cancer conditions, particularly cardiovascular disease (13), as reflected in the competing patterns of cardiovascular and cancer mortality in our analysis (Supplementary Figure S4). As a well-established causal risk factor for CVD, the management of LDL-C in cancer survivors has become increasingly important.

Previous studies in the general population have extensively examined the association between LDL-C and all-cause or cause-specific mortality, with many recent analyses reporting nonlinear relationships. A 2021 study based on NHANES including 19,034 participants (11) found a U-shaped association between LDL-C and all-cause mortality; however, due to the limited sample size, number of events, and duration of follow-up, no significant linear or nonlinear association was observed in the cancer subgroup. Similarly, a study in the Copenhagen General Population (14) reported that both low and high LDL-C levels were linked to increased all-cause mortality.

In comparison, cancer survivors are generally older and have a higher burden of chronic conditions. Emerging evidence indicates that LDL-C patterns in cancer survivors are distinct, influenced by tumor-driven lipid metabolism changes (15, 16), treatment effects (e.g., chemotherapy-induced lipid shifts) (17–19), and comorbid risk factors. Prior studies also suggest that cardiovascular risk differs between cancer survivors and the general population. In our cohort, the median LDL-C was 113 mg/dl (IQR: 90–138), lower than the general population median of 124 mg/dl, and the all-cause mortality curve was L-shaped (lowest risk at 119 mg/dl vs. 140 mg/dl U-shaped in non-cancer cohorts). Notably, survivors with LDL-C < 119 mg/dl experienced higher mortality, contrasting with newly diagnosed patients whose optimal LDL-C was higher (142 mg/dl), likely reflecting transient treatment effects (20–23). These findings highlight the need for cancer-specific LDL-C targets, particularly for long-term survivors, as generic thresholds may not adequately address their unique metabolic vulnerabilities and dynamic treatment-related changes.

Reverse causality may be a possible explanation for these findings. The lowest LDL-C quartile group (<90 mg/dl) in our study was characterized by older age and a higher prevalence of chronic diseases, including cardiovascular disease, hypertension, diabetes, and chronic kidney disease. Low LDL-C levels could, in part, be the outcome of poor health status and severe disease (24, 25). Compared to the general population (11, 14) and newly diagnosed cancer patients (4), individuals in our study exhibited a higher baseline age, greater disease burden, and elevated mortality rate (34.8% in our study vs. 21.3% in newly diagnosed cancer patients and 10.5% in the general population). Although sensitivity analyses that excluded individuals with chronic diseases at baseline and those who died within the first three years of follow-up consistently showed an association between low LDL-C levels and increased all-cause mortality, the impact of reverse causality cannot be entirely ruled out given the cancer-focused study design. After adjusting for confounding factors and accounting for potential reverse causality, the persistent association may largely reflect the impact of the underlying cancer. Notably, no significant direct association between LDL-C and cancer mortality was observed.

Due to differences in factors such as age, gender, race, and comorbidities, there is significant heterogeneity among cancer survivors. To examine the differences in all-cause mortality risk between low LDL-C and high LDL-C cancer survivors, stratified analyses were performed based on gender, age (<65 years or ≥65 years), race (White or non-White), BMI (<25, 25–30 or ≥30.00), smoking status (current smoker or non-smoker), cardiovascular diseases, diabetes, hypertension, and statin use. The association of higher LDL-C levels with lower all-cause mortality risk varied within subgroups, though the interaction between LDL-C and variables other than age in relation to all-cause mortality was not statistically significant. The pronounced effect observed primarily in the subgroup without pre-existing chronic conditions could be attributed to the fact that maintaining elevated LDL-C levels in cancer individuals with underlying diseases poses a greater risk for those conditions (24, 26). In contrast, among cancer individuals without such comorbidities, the effect of LDL-C is more likely to manifest in relation to cancer itself. Regarding the notable results in the subgroup not receiving statin therapy, a possible explanation is that cancer survivors undergoing statin treatment are inherently at higher risk (compared to those not receiving statins), despite having lower LDL-C levels (27, 28). A previous study in the general population identified a significant interaction between LDL-C levels and lipid-lowering treatments (14). However, in our study, this interaction was not observed, likely due to the limited number of cancer individuals using statins with LDL-C levels ≥119 mg/dl (*n* = 153). We found a significant interaction between age and LDL-C, where increasing age diminished the association of high LDL-C with reduced mortality but not reverse. This interaction effect was significant until age of 71.23 years. The age-stratified results showed that lower LDL-C levels were associated with a higher risk of all-cause mortality in both the group under 65 years and the group aged 65 years or older. Additionally, no nonlinear association between LDL-C levels and all-cause mortality was observed in the group under 65 years. This suggests association of LDL-C levels with all-cause mortality was complicated in elderly cancer survivors than in the young/middle-aged. This can be explained by a higher risk of long-term and late effects in adolescents and young adults (AYAs) cancer patients including infertility, sexual dysfunction, cardiovascular disease, and future cancers, compared with the elderly (29). In addition, an increasing body of evidence indicates that tumors in AYAs are molecularly distinct from those in both younger and older age groups, possibly suggesting differences in etiology and effective treatment (30, 31).

With the widespread adoption of early cancer screening and advances in cancer treatment, the survival duration of cancer patients has gradually increased. However, cancer patients are also facing additional health challenges, with cardiovascular disease (CVD) being the leading one (32). Our study provides new evidence for this trend and uncovers an interesting competitive relationship between cancer mortality and cardiovascular mortality. First, we found a “U-shaped” association between LDL-C levels and CVD mortality risk among cancer survivors overall, indicating that high LDL-C is associated with an increased risk of CVD, consistent with findings from the general population. Second, we observed an association between high LDL-C and higher all-cause mortality risk in cancer survivors undergoing secondary prevention for CVD, with further cause-specific cumulative incidence analysis showing that, at mid- to long-term follow-up (≥50 months), the cumulative incidence of CVD mortality in this subgroup exceeded that of cancer mortality. Lastly, in the ≥65 years age group, high LDL-C was also associated with higher all-cause mortality risk. Compared to the <65 years age group, the increase in CVD mortality risk was higher than the increase in cancer mortality risk. This age group had a higher proportion of CVD-related deaths, with more cancer survivors in the high or intermediate ASCVD risk categories. Previous studies have shown that the use of LDL-C-lowering drugs is associated with a reduction in mortality risk, and this effect is directly related to cholesterol reduction (33, 34). Overall, these findings suggest that the relationship between high LDL-C and all-cause mortality risk is influenced by CVD risk stratification and medical history, emphasizing the importance of evaluating and initiating interventions for high LDL-C cancer survivors with high CVD risk and known CVD history.

Current guidelines lack cancer-specific LDL-C management targets. While Chinese consensus (35) proposes tailored lipid monitoring based on chemotherapy toxicity, control thresholds remain tied to general ASCVD risk stratification (36, 37), neglecting cancer-specific factors or treatment history (38). Our findings underscore the inadequacy of population-derived LDL-C targets for survivors.

This study has several limitations. First, NHANES medical history data, including cancer status, alcohol use, smoking, and chronic diseases, are self-reported and may be subject to recall bias or inaccuracies. Second, LDL-C was measured only once at baseline, while serum levels can fluctuate over time; future studies should examine the relationship between dynamic LDL-C changes and mortality risk. Third, the dataset lacks detailed information on cancer stage and treatment, making it difficult to identify potential explanations for the L-shaped association between LDL-C and mortality beyond reverse causation. Future research should include long-term, repeated assessments in cancer survivors, considering cancer-specific and treatment-related factors, to better understand the mechanisms underlying the observed nonlinear associations between LDL-C and mortality.

In summary, our study observed a nonlinear association of LDL-C levels with all-cause and cardiovascular mortality in cancer survivors, wherein low levels of LDL-C were associated with an increased mortality risk, and the identified optimal LDL-C thresholds were 119 mg/dl for all-cause mortality and 124 mg/dl for cardiovascular mortality. Inverse LDL-C-mortality association particularly in cancer survivors <65 years and without prior CVD. These results enhanced the understanding of the association between LDL-C levels and all-cause, cardiovascular, and cancer mortality within this population, emphasize the prognostic role of LDL-C in cancer survivors and suggest that tailored LDL-C management targets may be required for this population.

## Data Availability

Publicly available datasets were analyzed in this study. This data can be found here: https://www.cdc.gov/nchs/nhanes/.
