# Supplementary material for: L-shaped association of low-density lipoprotein cholesterol with all-cause and cardiovascular mortality in cancer survivors: a population-based cohort study
Source: Front Cardiovasc Med. 2025 Sep 15;12:1593824. doi: 10.3389/fcvm.2025.1593824 (PMC12477126; doi:10.3389/fcvm.2025.1593824)
Supplement: Supplementary file 1 [file Supplementaryfile1.pdf]

Content of Supplementary Materials

Supplementary figure legends **2**

Figure 1 Flowchart of LDL-C cohort 3

Figure 2 The Number and Percentage of Missing Data 4

Figure 3 Adjusted Hazard Ratios (95% CIs) between LDL-C and All-cause, Cardiovascular and Cancer Mortality in Cancer Individuals 5

Figure 4 Cumulative Incidence of Cardiovascular Diseases and Cancer 6

Figure 5 Association of LDL-C with All-cause Mortality Stratification by Age 7

Figure 6 Age-Stratified Proportional Distribution of Mortality Causes 8

Figure 7 Age-Stratified Distribution of ASCVD Risk Categories in Cancer Survivors 9

Figure 8 Penalized Smoothing Splines Evaluating the Nonlinear Association of LDL-C Levels with All-Cause and Cardiovascular Mortality in Cancer Individuals. 10

Figure 9 Sensitivity Analyses 11

Weights in NHANES **12**

Selecting the correct weight in NHANES 12

Constructing Weights for Combined NHANES Survey Cycles 12

Covariates **13**

Correction for Serum Creatinine **15**

Supplementary figure legends

Figure 1 Flowchart of LDL-C cohort.

Figure 2 The Number and Percentage of Missing Data.

Figure 3 Adjusted Hazard Ratios (95% CIs) between LDL-C and All-cause, Cardiovascular and Cancer Mortality in Cancer Individuals.

Figure 4 Cumulative Incidence of Cardiovascular Diseases and Cancer

Figure 5 Association of LDL-C with All-cause Mortality Stratification by Age

Figure 6 Age-Stratified Proportional Distribution of Mortality Causes

Figure 7 Age-Stratified Distribution of ASCVD Risk Categories in Cancer Survivors

Figure 8 Penalized Smoothing Splines Evaluating the Nonlinear Association of LDL-C Levels with All-Cause and Cardiovascular Mortality in Cancer Individuals.

Figure 9 Sensitivity Analyses

Figure 1 Flowchart of LDL-C cohort

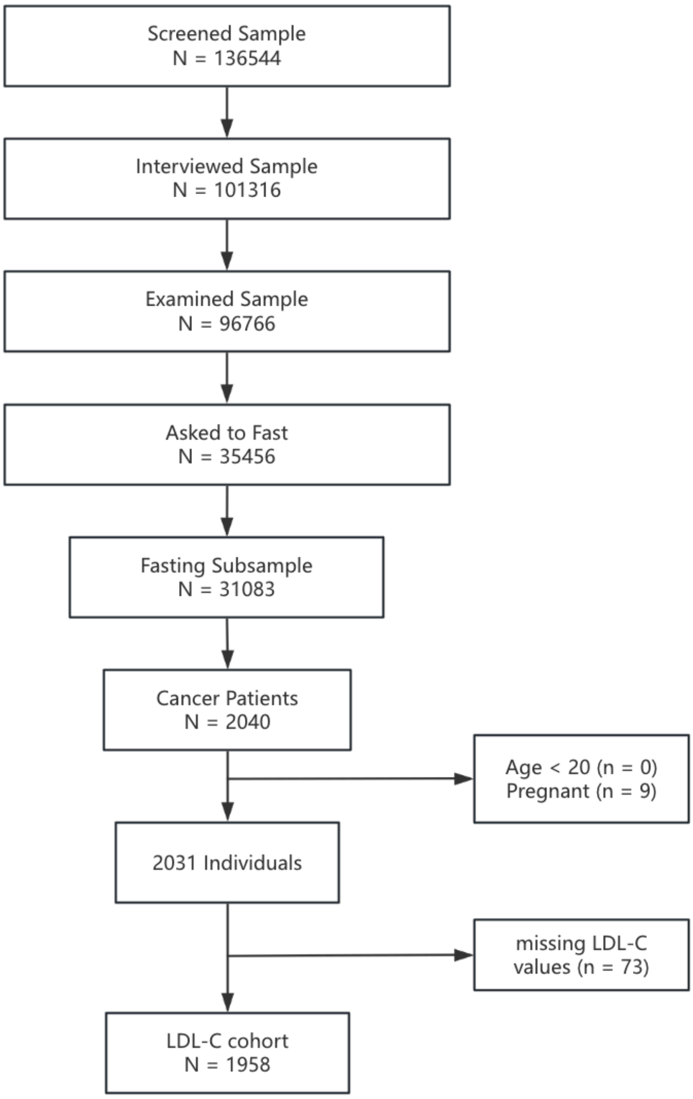

Figure 2 The Number and Percentage of Missing Data

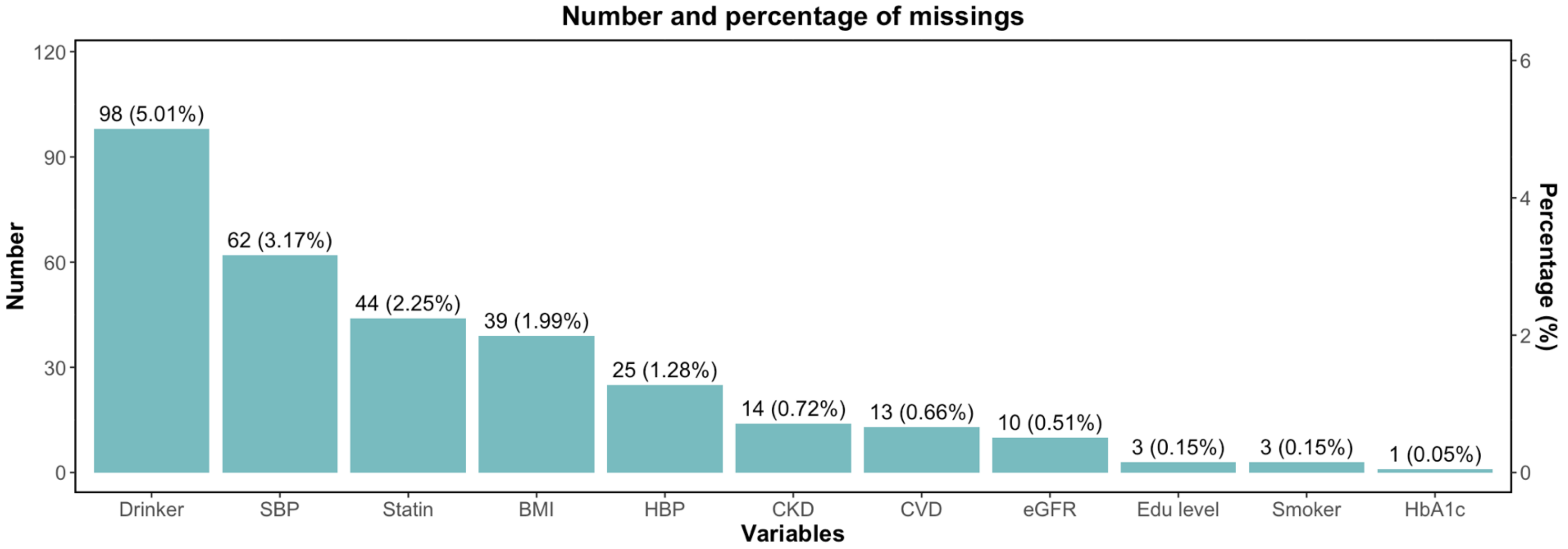

Drinker = drinking status; SBP = systolic blood pressure; Statin = statin use; BMI = body mass index; HBP = hypertension; CKD = chronic kidney disease; CVD = cardiovascular disease; eGFR = estimated glomerular filtration rate; Edu level = education level; Smoker = smoking status; HbA1c = glycated hemoglobin.

Figure 3 Adjusted Hazard Ratios (95% CIs) between LDL-C and All-cause, Cardiovascular and Cancer Mortality in Cancer Individuals.

All-cause Mortality

Group	Individuals	Events	Person years	Event rate per 1000 person years	Age and gender adjusted hazard ratio (95% CI)	Multivariable adjusted hazard ratio (95% CI)
Q1(<90mg/dL)	552	213	3786.9	56.2	1.42 (1.07 to 1.88)	1.50 (1.15 to 1.96)
Q2(90-113mg/dL)	487	169	3885.3	43.5	1.24 (0.95 to 1.61)	1.25 (0.95 to 1.63)
Q3(113-138mg/dL)	492	160	4251.5	37.6	Reference	Reference
Q4(≥138mg/dL)	427	139	3662.4	38.0	1.05 (0.78 to 1.43)	1.02 (0.74 to 1.39)

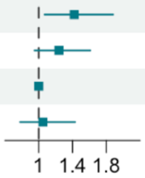

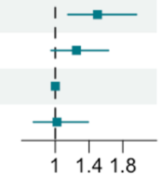

Cardiovascular Mortality

Group	Individuals	Events	Person years	Event rate per 1000 person years	Multivariable adjusted cause-specific hazard ratio (95% CI)	Multivariable adjusted subdistribution hazard ratio (95% CI)
Q1(<90mg/dL)	552	69	3786.9	18.2	2.40 (1.36 to 4.25)	2.23 (1.36 to 3.65)
Q2(90-113mg/dL)	487	40	3885.3	10.3	1.45 (0.79 to 2.63)	1.53 (0.92 to 2.54)
Q3(113-138mg/dL)	492	27	4251.5	6.4	Reference	Reference
Q4(≥138mg/dL)	427	39	3662.4	10.6	1.43 (0.79 to 2.58)	1.75 (1.06 to 2.90)

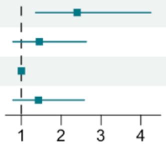

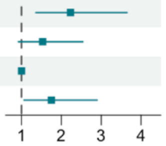

Cancer Mortality

Group	Individuals	Events	Person years	Event rate per 1000 person years	Multivariable adjusted cause-specific hazard ratio (95% CI)	Multivariable adjusted subdistribution hazard ratio (95% CI)
Q1(<90mg/dL)	552	60	3786.9	18.2	1.51 (1.00 to 2.27)	1.01 (0.68 to 1.50)
Q2(90-113mg/dL)	487	46	3885.3	10.3	1.15 (0.73 to 1.80)	0.90 (0.60 to 1.35)
Q3(113-138mg/dL)	492	52	4251.5	6.4	Reference	Reference
Q4(≥138mg/dL)	427	48	3662.4	10.6	1.07 (0.67 to 1.69)	1.09 (0.73 to 1.63)

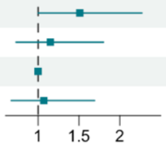

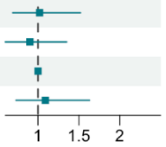

Figure 4 Cumulative Incidence of Cardiovascular Diseases and Cancer

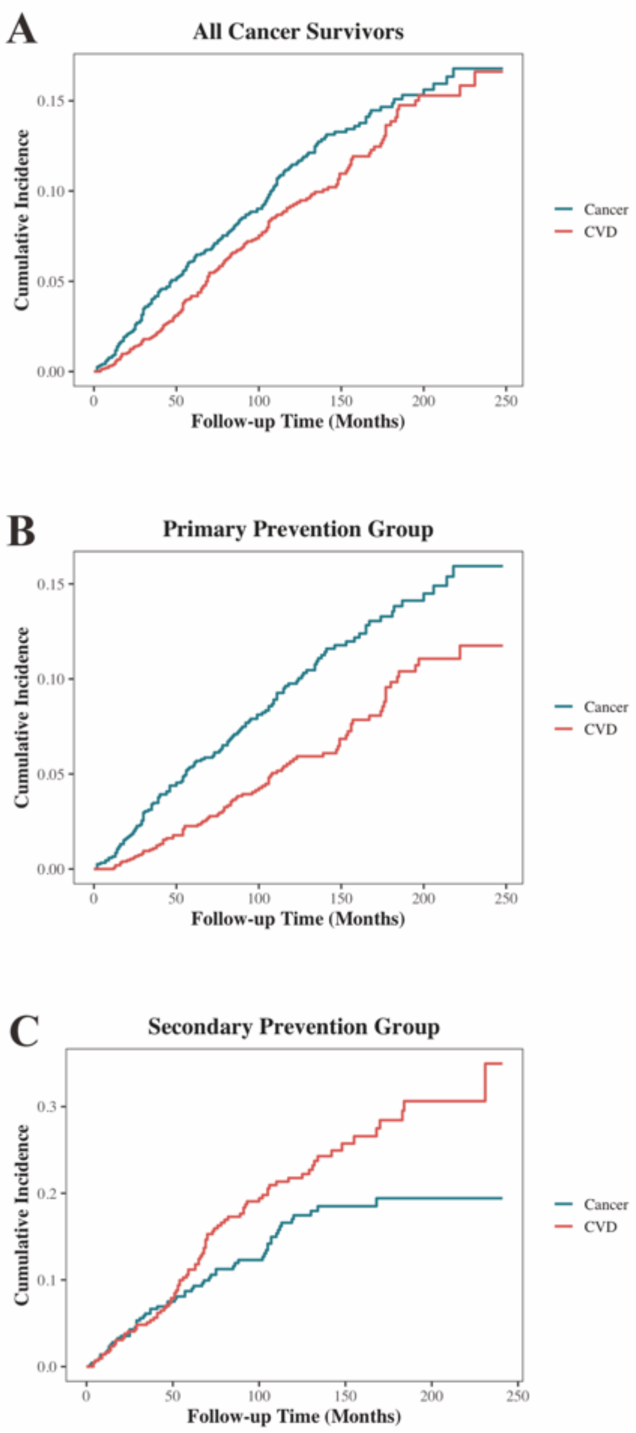

Figure 5 Association of LDL-C with All-cause Mortality Stratification by Age

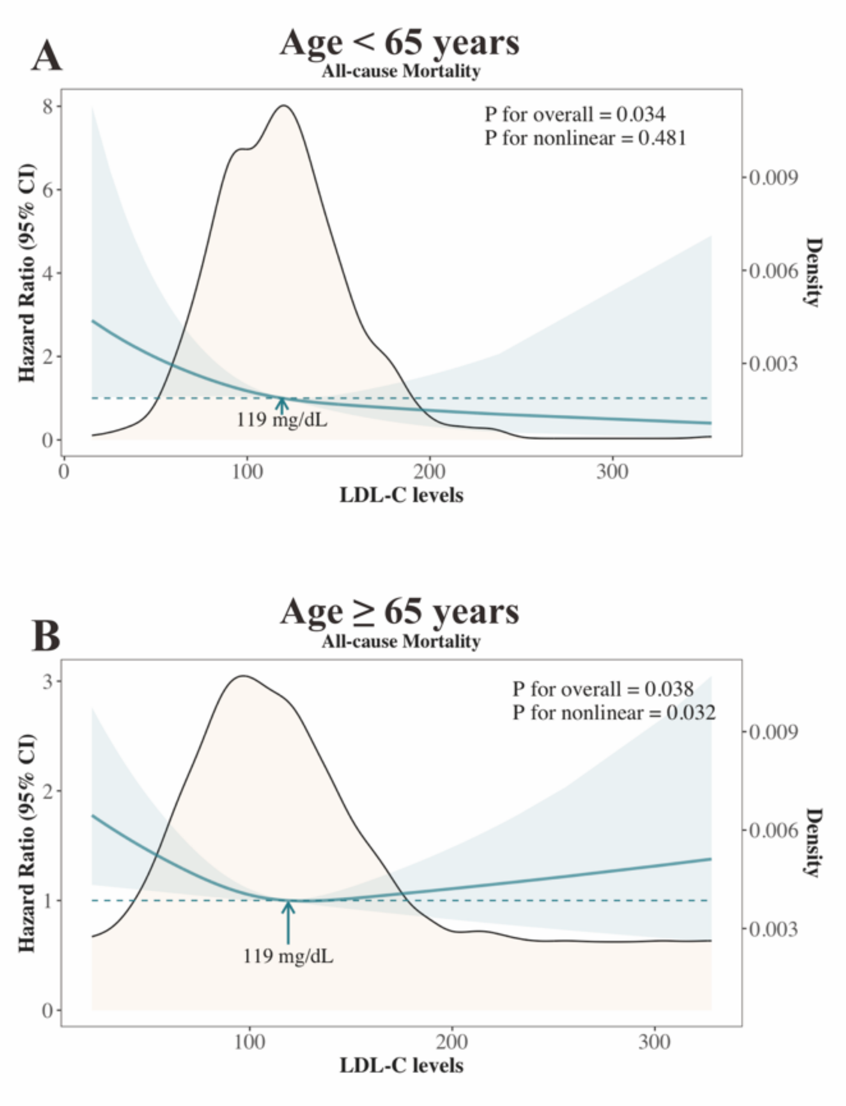

Figure 6 Age-Stratified Proportional Distribution of Mortality Causes

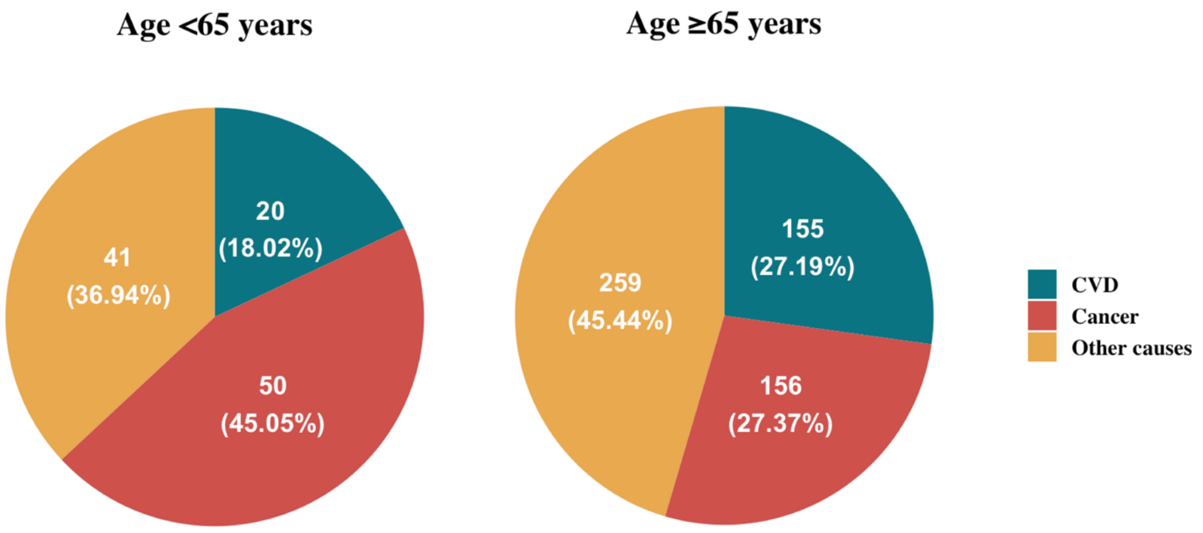

Figure 7 Age-Stratified Distribution of ASCVD Risk Categories in Cancer Survivors

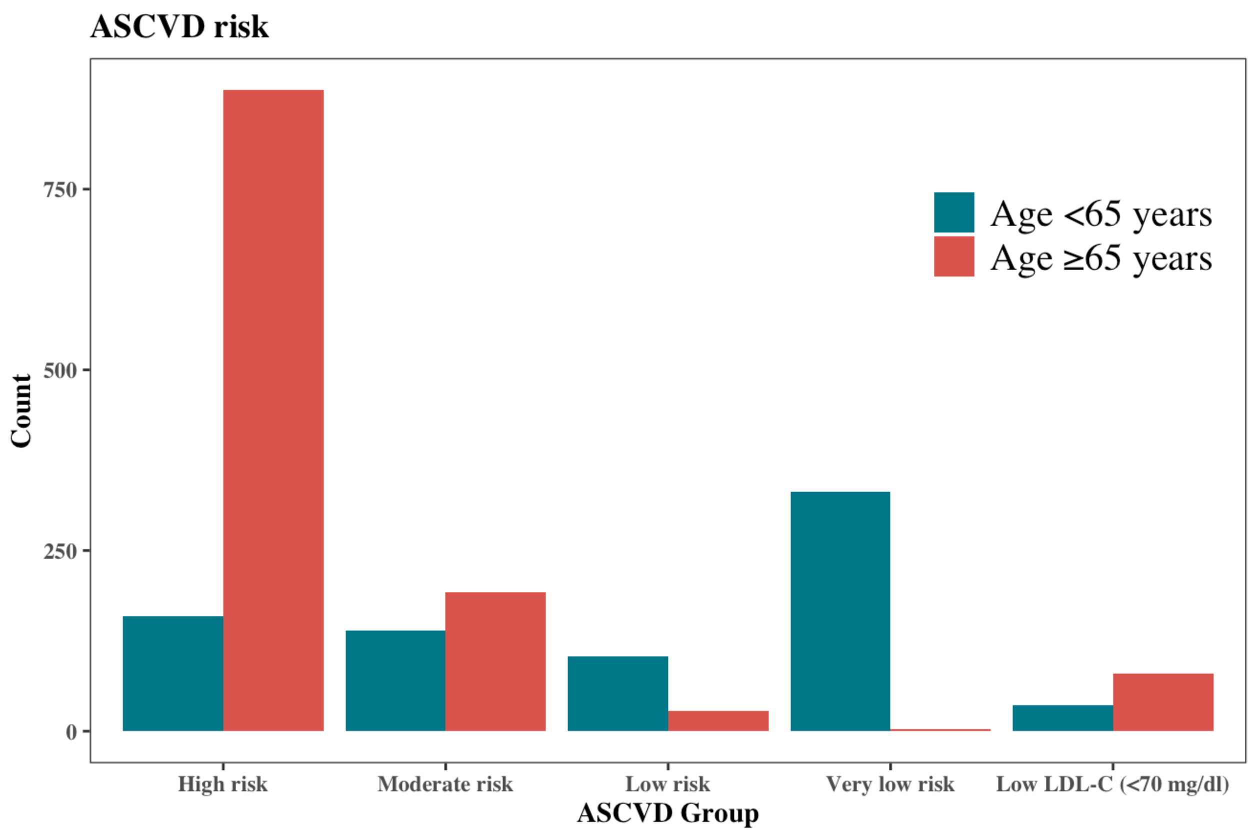

Figure 8 Penalized Smoothing Splines Evaluating the Nonlinear Association of LDL-C Levels with All-Cause and Cardiovascular Mortality in Cancer Individuals.

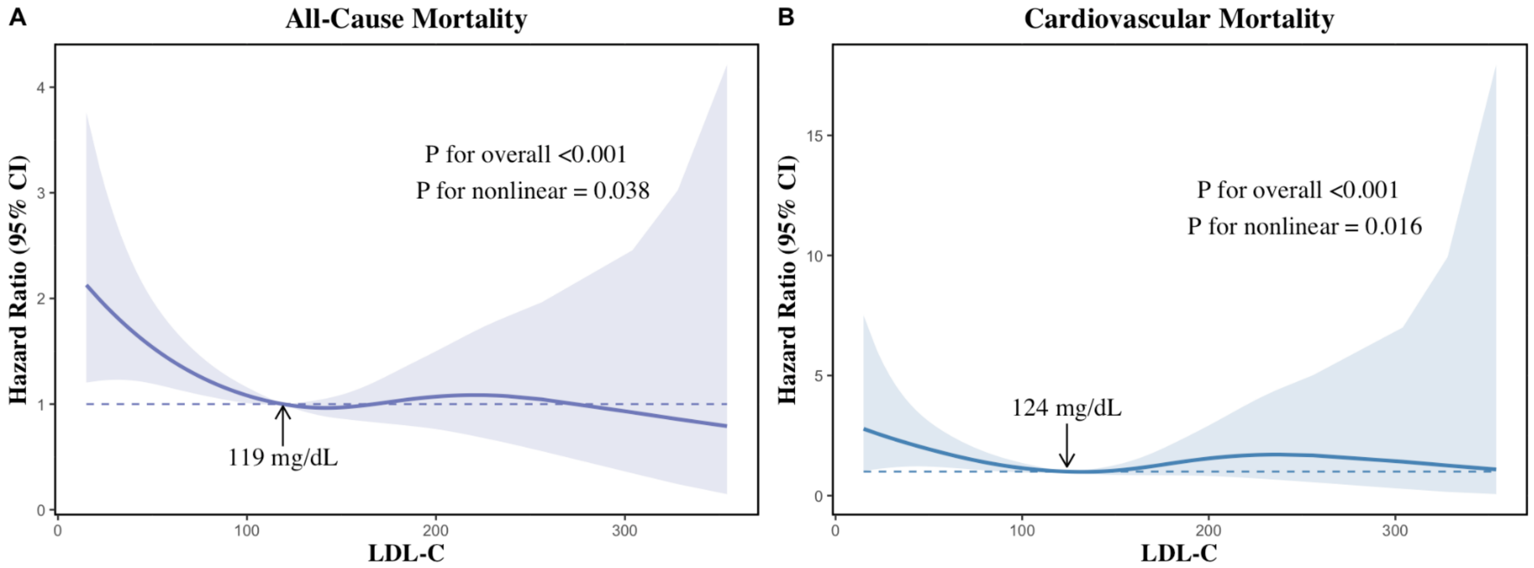

Solid lines represent adjusted hazard ratios for A) all-cause mortality and B) cardiovascular mortality, with shaded areas indicating 95% confidence intervals derived from penalized smoothing splines (df = 3). Arrows indicate the inflection points of LDL-C derived from threshold effect analyses. Analyses were adjusted for gender, age, race, education level, smoking status, body mass index, systolic blood pressure, eGFR, cardiovascular disease, diabetes, statin use and cancer type.

Figure 9 Sensitivity Analyses

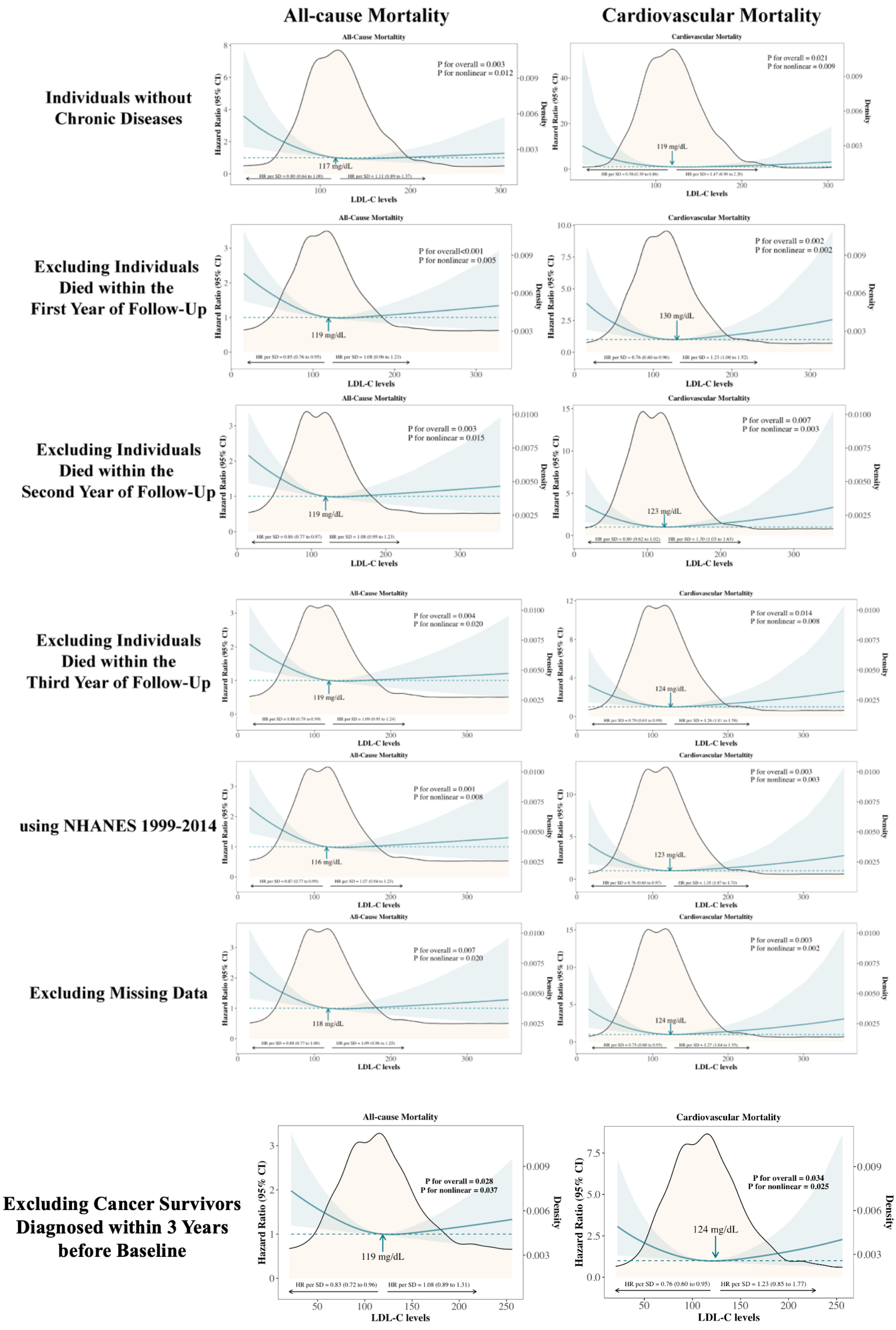

Weights in NHANES

Weights are created in NHANES to account for the complex survey design (including oversampling), survey non-response, and post-stratification adjustment to match total population counts from the Census Bureau. Our study followed the official NHANES tutorials to select the appropriate types of weights and considered these weights in the merging and analysis of the data.

Selecting the correct weight in NHANES

The principle for selecting weights is to use the weight of the smallest subpopulation that includes all the variables in the analysis. Since we only included participants with LDL-C data who fasted for at least 8.5 hours before the blood test, and this group is approximately half the sample of those who were examined in MEC, we selected the fasting subsample weights (WTSAF4YR or WTSAF2YR). NHANES creates fasting weights (WTSAF2YR) for participants in each data cycle. WTSAF4YR is the combined fasting weight for 1999-2002 NHANES cycles.

Constructing Weights for Combined NHANES Survey Cycles

The sample design for NHANES makes it possible to combine two or more survey cycles to increase the sample size and analytic options. Each two-year cycle and any combination of two-year cycles is a nationally representative sample. According to NHANES tutorials, we constructed sample weight for NHANES 1999-2018 as described below:

$$\text{For NHANES 1999 – 2002: weight} = \text{WTSAF4YR} * 2/10$$

$$\text{For NHANES 2003 – 2018: weight} = \text{WTSAF2YR} * 1/10$$

Covariates

Age, sex, race-ethnicity, education, smoking status and drinking status were self-reported by participants. Educational level was recoded into three categories based on questionnaire responses: less than high school, high school or equivalent, and college or above. Drinkers were defined as participants who drank at least 12 alcohol drinks in any given year. Moderate versus heavy drinkers were defined as participants who consumed <1 versus ≥1 drink/day. Smokers were defined as participants who reported smoking at least 100 cigarettes during their lifetime, with former smokers defined as participants who reported smoking at least 100 cigarettes, but not currently smoking. Body mass index (BMI) was calculated as weight (kilograms) divided by squared height (meters2).

Lipid measurements other than LDL-C (including TG, HDL-C, TC) and HbA1c were obtained from laboratory tests. eGFR was calculated based on age, gender, and serum creatinine using the 2021-CKD-EPI equation(19). Due to changes in the location, equipment, and methods of serum creatinine testing, adjustments were made to the serum creatinine values for the NHANES 1999-2000 and NHANES 2005-2006 cycles to ensure comparability across cycles (**Supplementary Materials - Correction for Serum Creatinine**).

Cardiovascular diseases (CVD) were a combination of self-reported doctor diagnosed angina, coronary heart disease, heart attack or stroke, Diabetes was defined as self-reported doctor diagnosed diabetes or HbA1c ≥ 6.5%. Hypertension

was defined as systolic blood pressure (SBP) ≥ 140 mmHg, diastolic blood pressure (DBP) ≥ 90 mmHg, or taking blood pressure control medication. Chronic kidney disease was defined as self-reported doctor diagnosed weak kidney or eGFR < 60.

Statin users were identified based on a prescription medications questionnaire, which collected data on the use of prescription medications during the one-month period prior to the survey. Non-statin users were defined as those who did not report statin use on the prescription medications questionnaire or who did not self-report doctor diagnosed high cholesterol level, being advised to take medication, or currently taking medication for cholesterol.

Correction for Serum Creatinine

Due to differences in measurement methods, instruments, and locations, it is necessary to adjust serum creatinine values across different cycles of NHANES to ensure comparability. According to the NHANES analytic notes, we used the following formula to adjust the serum creatinine values

For NHANES 1999 – 2002:

$$\text{Standard Creatinine } (\text{Y}, \text{mg/dL}) = 1.013 * \text{NHANES Creatinine } (\text{X}) + 0.147\,.$$

For NHANES 2005 – 2006:

$$\text{Standard creatinine } (\text{Y}, \text{mg/dL}) = -0.016 + 0.978\ \text{X}\ (\text{NHANES } 05 - 06 \text{ uncalibrated serum creatinine}, \text{mg/dL})$$

No correction is necessary for serum creatinine values in other NHANES cycles.
